# Envenomation Leads to Venom Protein Reduction and a Recovery Period in Bumblebee Workers

**DOI:** 10.1002/ece3.74629

**Published:** 2026-11-05

**Authors:** Safira Moog, Mario Dejung, Amitkumar Fulzele, James C. Carolan, Thomas J. Colgan

**Affiliations:** ^1^ Institute of Organismic and Molecular Evolution Johannes Gutenberg University Mainz Germany; ^2^ Institute of Molecular Biology (IMB) Mainz Germany; ^3^ Department of Biology Maynooth University Maynooth Co. Kildare Ireland

**Keywords:** bumblebee, envenomation, proteomics, venom

## Abstract

The evolution and deployment of venom systems in animals have been central to their ecological success, though not without costs. For social bees, venom plays a key role in nest defence; yet for species such as honeybees, the act of stinging generally leads to death. However, for other social bee species such as bumblebees, where stinging is not apparently fatal, it raises the question of what, if any, are the consequences associated with venom usage (envenomation). Here, we investigated proteomic changes to understand consequences that can occur at the molecular level in the venom sac, the primary reservoir for venom storage, of *Bombus terrestris* workers over a short time‐course post‐envenomation. Our analysis reveals three key findings: (1) envenomation leads to changes in the venom sac proteome, with affected proteins generally reduced in the bumblebee venom sac, suggestive of a molecular cost; (2) quantified reductions of traditional venom‐associated proteins post‐envenomation indicate changes in individual venom protein abundance post‐usage; and (3) recovery period for bumblebee post‐envenomation to return to a non‐envenomation state was required, further indicating a potential cost at the molecular level associated with venom usage. Overall, our findings provide new insights into the molecular consequences of venom usage in bumblebees.

## Graphical abstract

The evolution and deployment of venom systems in social bees have been central to their ecological success, though not without costs. However, for species such as bumblebees, where stinging is not apparently fatal, it raises the question of what, if any, are the consequences associated with venom usage (envenomation). Here, we investigated proteomic changes in the venom sac, finding envenomation leads to changes in the venom sac proteome, with affected proteins generally reduced in the bumblebee venom sac, suggestive of a molecular cost.

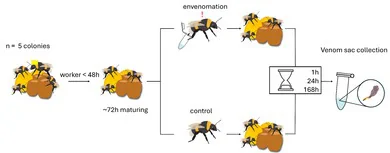

## 1 Introduction

Predation and competition place selective pressures on individuals that lead to adaptive strategies and traits to enhance survival (Abrams 2000). One such trait is venom, an evolutionary innovation, which has evolved in numerous organisms to aid in predation, as well as in defence, acting as a strong deterrent against predators and nuisances through inducing pain, paralysis, and even death (Casewell et al. 2013; Schendel et al. 2019). Venoms are highly complex mixtures of bioactive compounds containing a high number of small peptides, proteins, and toxins, as well as polyamines, organic molecules, and salts (Fry et al. 2009). Venomous animals actively inject their self‐synthesised venom into targets through the process of envenomation, disrupting normal physiological or biochemical processes (Fry et al. 2009), serving key roles in predation and defence. The evolutionary and ecological importance of venom is underlined by its independent evolution at least 101 times across diverse animal lineages (Schendel et al. 2019).

Although venom use provides clear advantages in predation and defence, maintaining and using the venom system can be energetically costly, both in terms of product synthesis as well as post‐use regeneration. For example, increases in metabolic rate have been described in snakes, spiders, and scorpions during venom regeneration (McCue 2006; Nisani et al. 2007, 2012), potentially taking several days, such as 16 days in the spider *Cupiennius salei* (Boevé et al. 1995) and up to 28 days in snakes (Morgenstern and King 2013). This suggests that venom usage is energetically costly and, therefore, should not be used wastefully. Venomous animals have evolved strategies favouring conservative venom use through physiological, morphological, and behavioural adaptations to minimise costs, as postulated in the venom optimisation hypothesis (Wigger et al. 2002). An increasingly recognised concept supporting the venom optimisation hypothesis is that venom use can be regulated depending on the ecological context (Schendel et al. 2019; Morgenstern and King 2013). The state of the envenomating organism and that of the prey are both suggested to play a role in venom use as venom regulation can be affected by prey size and prey type. For example, the spider *C. salei* alters the amount of venom it injects depending on the size of the prey likely to minimise venom use (Wigger et al. 2002). Furthermore, from an evolutionary perspective, several secondary losses or reductions of morphological traits associated with venom synthesis or delivery have occurred, such as in a marbled sea snake caused by a dietary switch (Li et al. 2005), implying that venom is energetically costly to maintain at an evolutionary scale (Morgenstern and King 2013). While energetic expenditure by predators could be compensated by the consumption of prey (Morgenstern and King 2013), there is no such direct compensation in terms of reward for venom usage during defence. Therefore, animals using defensive venoms may be exposed to stronger selection pressures on venom regulation than species using venom primarily for predation, as failure of defence can lead to potential death (Schendel et al. 2019). This may limit the evolution of venom regulation mechanisms and may be associated with particularly high costs in terms of usage. Beyond defence, venom serves multiple functions, including prey capture, external immunity, and pathogen control. The antimicrobial properties of certain venom components also contribute to protection against infections. In social insects, including ants, some bees and wasps, venom has been shown to play additional roles in colony hygiene by disinfecting nests or in external immunity by venom components being deposited on the cuticle (Baracchi et al. 2011, 2012; Moreau 2013).

An ecologically and biomedically relevant group that employs venom in defence are bees (Clade Anthophila), a taxon‐rich group (20,000 described species (Michener 2007)) of important ecological and commercial pollinators. While venom usage by bees is predicted to have evolved initially for hygienic purposes, such as in the sterilisation of nesting sites (Moreau 2013; Stöcklin et al. 2010), social bee venoms are generally associated with defence, causing pain, tissue damage, and potentially death (Koludarov et al. 2023). As in other Hymenoptera, the venom system of bees evolved from an ancestral ovipositor and accessory sex glands. Thus, the venom system is only present in females while males lack the apparatus and are unable to sting (Robertson 1968; Bridges and Owen 1984). Despite the importance of venom, venom usage in bees has largely been studied from a biomedical perspective, with our knowledge of venom use in an ecological context being limited. The best studied bee species, the western honeybee *Apis mellifera*, highlights certain costs placed on individuals in terms of self‐sacrifice during nest defence. In honeybees, morphological adaptations and associated defence tactics have evolved to maximise pain in vertebrate threats, leading to a high individual cost in the form of fatality after stinging through autotomy (self‐amputation) of the stinging apparatus (Hermann 1971). However, such morphological adaptations are not present in other stinging bees, such as bumblebees, which do not possess the same modified barb found in honeybee workers. This allows bumblebees to repeatedly sting both invertebrate and vertebrate threats (Breed et al. 2004; Steen et al. 2005). Compared to other social bees, such as honeybees, which may number in the thousands, bumblebee colonies are considerably smaller (~150–300 workers (Alford 1975)), which means workers may be of greater individual importance to colony productivity and defence. Moreover, bumblebees show a range of context‐dependent behavioural repertoires, ranging from avoiding conflicts by flight to threatening behaviour, such as buzzing, hissing, biting or leg movement (Free 1958; Kirchner and Röschard 1999), although such behavioural repertoires have been shown to commonly precede stinging (Varnon et al. 2021), highlighting its importance as a defence against predators and possible intruders. However, our understanding of potential molecular costs associated with venom usage within bumblebee workers is currently unknown. Such molecular costs may be reflected in changes in venom protein abundance, placing energetic demands for regeneration, as well as limiting reuse or potency of venom.

To understand whether envenomation results in changes in bumblebee venom protein abundance, as well as venom regeneration time, we employed mass spectrometry‐based proteomics to investigate changes in the venom sac proteome of *Bombus terrestris* workers over a short time‐course (1, 24, 168 h) after envenomation. We chose the venom sac as it acts as a reservoir for the storage of venom proteins that are primarily produced in the connected tubular venom glands (Roat et al. 2006). Therefore, protein changes in the venom sac proteome can provide insight into the molecular response and recovery post‐envenomation. As venom regeneration is potentially energetically costly (Morgenstern and King 2013; Wigger et al. 2002; Evans et al. 2019), we predict that envenomation leads to: (1) proteomic changes in the venom sac (referring to the venom sac tissue and the venom contained therein), including (2) reduced abundances of known venom proteins; and (3) a period of reduced abundance of venom‐associated proteins compared to non‐envenomation levels of control bees.

## 2 Material and Methods

### 2.1 Colony Maintenance

*Bombus terrestris* colonies (*n* = 5) were obtained from Koppert Biological Systems (Netherlands) and kept under constant conditions at 28°C and under red light illumination. Sugar water (Apiinvert, Germany) and pollen (Biobest, Belgium) were provided *ad libitum*. For each source colony, callows (distinguishable from older workers due to brighter colouration, and folded‐back wings) were collected within 48 h upon eclosion. We used callows to control for potential effects of age. Upon collection, these callows were then transferred to a randomly assigned treatment box (either control or envenomation; 15 cm × 15 cm × 15 cm) where they were kept for approximately 72 h to allow for maturation (Colgan et al. 2011). Prior to the introduction of the callows, each treatment box also contained brood and sugar water, as well as four related mature workers (distinguishable due to clipped wings) to simulate a colony‐like setting.

### 2.2 Envenomation Procedure

To induce envenomation, we used parafilm‐covered 0.2 mL tubes filled with 100 μL autoclaved phosphate buffered saline (PBS) to collect venom from each worker. For each collection, we temporarily restrained the bee and placed the collection tube close to the aculeus. We visually confirmed stinging by the presence of a hole in the parafilm. For control bees, they experienced the same handling procedure, but no venom was collected. Post‐envenomation, workers were returned to their boxes and remained until a designated sampling time point, which was either 1, 24, or 168 h (1 week) after procedure (Figure 1). At each designated collection time point, workers were transferred into individual cryotubes, snap‐frozen in liquid nitrogen, and stored at −80°C before being dissected. In total, we collected 25 workers (Envenomation group: 1 h (*n* = 5), 24 h (*n* = 4), and 168 h (1 week, *n* = 3); Control group: 1 h (*n* = 5), 24 h (*n* = 4), and 168 h (*n* = 4)). Individuals, one each from a different colony, were used for each treatment group and time point. We also assessed the collected venom for protein content using the Qubit protein assay kit (Q3321; Invitrogen, Germany).

**FIGURE 1:**
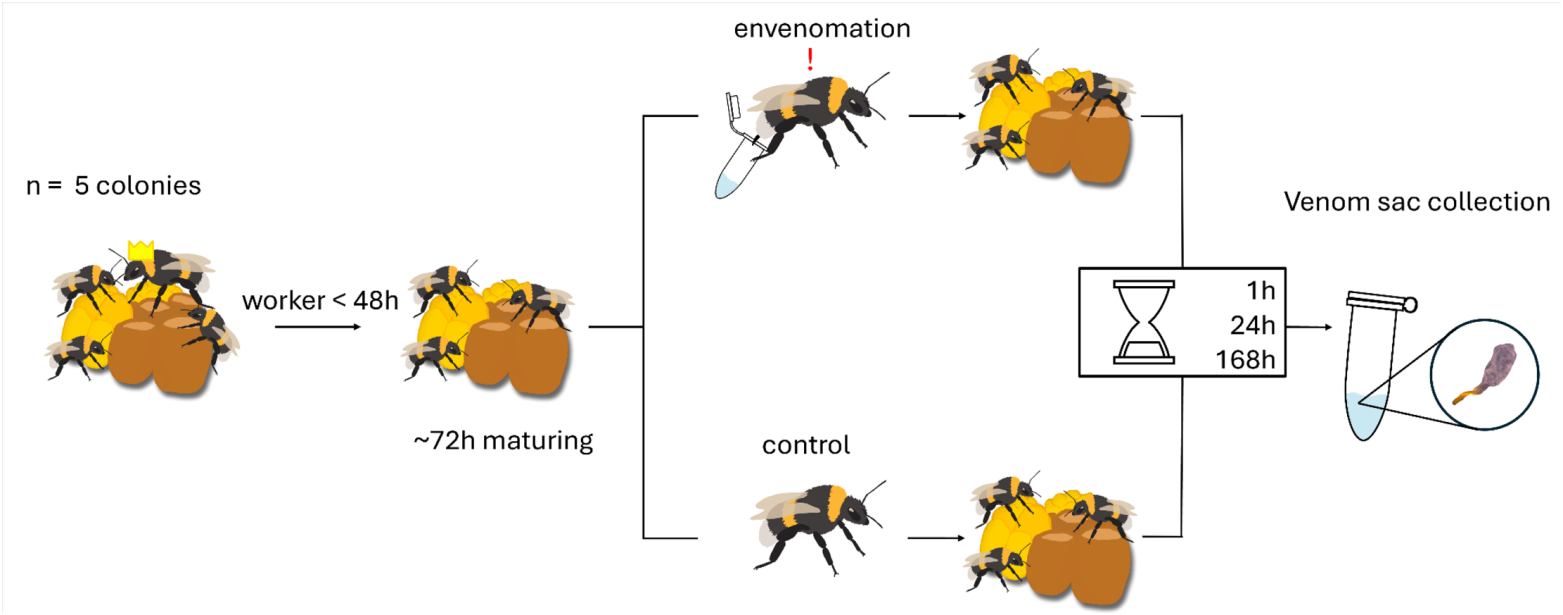
Envenomation procedure and venom sac collection. Worker callows (< 48 h after eclosion) of five different colonies were collected and kept for 72 h under colony like conditions until the envenomation and control treatment was performed. In the envenomation treatment, a bee stung into a parafilm‐covered tube while control bees did not sting. The bees were returned to a nursery colony and sampled at 1, 24 and 168 h, post‐treatment. The venom sac was later dissected and analysed using mass‐spectrometry‐based proteomics.

### 2.3 Venom Sac Extraction and Proteomic Analysis

For each bee, the venom sac tissue and the fluid within the venom sac were extracted while associated glands, the venom gland and the Dufour's gland, were removed. Subsequently, the venom sac was homogenised in PBS. To determine differently abundant proteins, we performed mass spectrometry.

Protein quantity of venom sac samples was measured using the same Qubit protein assay kit as above, and 5 μg protein per sample was resolved briefly in a 4%–12% NuPAGE Bis‐Tris gradient gel, stained with Coomassie blue and cut into small gel cubes, followed by destaining in 50% ethanol/25 mM ammonium bicarbonate. Afterwards, proteins were reduced in 10 mM DTT at 56°C and alkylated by 50 mM iodoacetamide in the dark at room temperature. Enzymatic digestion of proteins was performed using trypsin in 50 mM ammonium bicarbonate at 37°C overnight. Following peptide extraction sequentially using 30% and 100% acetonitrile, the sample volume was reduced in a centrifugal evaporator to remove residual acetonitrile. The peptide solution was purified by solid phase extraction in C_18_ StageTips.

Peptides were separated on an EASY‐nLC 1000 system (Thermo Scientific) via an in‐house packed 30‐cm analytical column (inner diameter: 75 μm; ReproSil‐Pur 120 C_18_, Dr. Maisch GmbH) by online reverse phase chromatography through a 105‐min non‐linear gradient of 1.6%–32% acetonitrile with 0.1% formic acid at a nanoflow rate of 225 nL/min. The eluted peptides were sprayed directly by electrospray ionisation into a Q Exactive Plus Orbitrap mass spectrometer (Thermo Scientific). Mass spectrometry was conducted in data‐dependent acquisition mode using a top10 method with one full scan (scan range: 300 to 1650 m/z; resolution: 70,000, target value: 3 × 10, Dr. Maisch GmbH) by online reverse phase chromatography through a 105‐min non‐linear gradient of 1.6%–32% acetonitrile with 0.1% formic acid at a nanoflow rate of 225 nL/min. The eluted peptides were sprayed directly by electrospray ionisation into a Q Exactive Plus Orbitrap mass spectrometer (Thermo Scientific). Mass spectrometry was conducted in data‐dependent acquisition mode using a top10 method with one full scan (scan range: 300 to 1650 m/z; resolution: 70,000, target value: 3 × 10^6^, maximum injection time: 20 ms) followed by 10 fragment scans via higher energy collision dissociation (HCD; normalised collision energy: 25%, resolution: 17,500, target value: 1 × 10^5^, maximum injection time: 120 ms, isolation window: 1.8 m/z). Precursor ions of unassigned or +1 charge state were rejected. Additionally, precursor ions already isolated for fragmentation were dynamically excluded for 20s.

MS/MS data were analysed using MaxQuant (v.1.6.10.43; (Cox and Mann 2008)) with match between runs activated. Mass spectra were searched against a target‐decoy database containing the forward and reverse sequences of *B. terrestris* proteome predicted from the genome assembly (Bter_1.0; (Sadd et al. 2015)) obtained from Ensembl Metazoa release 53, as well as a default list of contaminants. As certain venom‐associated proteins, such as bombolitin, have truncated gene models within the *B. terrestris* genome assembly, we included proteins from two melittin homologues in *Bombus impatiens* . MaxLFQ algorithm was employed for protein quantification, using its default normalisation option and requiring a minimum LFQ (label‐free quantification) ratio count of one. Detected proteins were filtered for reverse binders and contaminants. MaxQuant classified a protein as expressed when at least one unique peptide for each protein was detected or when a single peptide was identified across multiple samples by multiple MS/MS spectra in the same matching group (six matching groups in total: one group per treatment and time point). Missing values were imputed in two route steps. Proteins not detected in a single replicate were imputed with a normal distribution at mean of the measured replicates. If a protein was not detected in more than one sample, then the values were imputed close to the detection limit.

### 2.4 Comparison With Previous Venom Proteomes

As known venom‐associated proteins would be expected to be detected in our venom sac analysis, we compared a list of identified proteins in our dataset with venom associated proteins previously described for *B. terrestris* (Van Vaerenbergh et al. 2015). To ensure high confidence in previously identified venom‐associated proteins, we downloaded data from Van Vaerenbergh et al. (2015) and filtered identified proteins based on high peptide‐spectrum matches (PSM value ≥ 20), resulting in the retention of 43 high‐confidence venom‐associated proteins (Supporting Information, Table S1). We then examined the presence of these proteins in our venom sac proteome dataset to identify their presence, as well as examine if their abundance changed post‐envenomation. In total, across all samples, the venom sac proteome contained 2031 proteins, including 28 previously described *B. terrestris* venom proteins (Van Vaerenbergh et al. 2015) (Supporting Information, Table S1).

### 2.5 Principal Component Analysis and Differential Abundance Analysis

To examine differences in proteome profiles between treatments and across time points, we first performed a principal component analysis (PCA) using only proteins that had non‐imputed log‐transformed LFQ intensity values. To identify differentially abundant proteins between treatments at each time point, we implemented two approaches. First, we fitted a linear model (LM) for each protein using R (v. 4.3.3; (R Core Team 2024)) and the R package limma (v. 3.58.1; (Ritchie et al. 2015)). Imputed LFQ values of envenomation and control bees were included as the response variable while treatment, time, as well as their interaction, were included as fixed effects. As part of the limma‐based analysis, empirical Bayes moderation was applied to the linear models, and contrasts were tested to assess differences in protein expression between envenomation and control groups. *p*‐Values were corrected for multiple testing using the Benjamini–Hochberg method and proteins with an adjusted *p*‐value < 0.05 were considered statistically significant. Identified differently abundant proteins changing due to time are presented in the Supporting Information (Supporting Information, Figure S1). As an additional approach, we performed pairwise Welch's *t*‐tests to compare treatments at each time point using Perseus (Tyanova et al. 2016), a software tool commonly used for the analysis of MaxQuant‐generated output. *p*‐Values were corrected for multiple testing in R using a false discovery rate of 0.05 (Benjamini‐Hochberg method) (Supporting Information). Both approaches identified a similar number of enriched proteins between envenomation and control bees with a high level of overlap of differentially expressed proteins identified by both limma and Perseus (Percentage of identified proteins by Perseus that were also identified by limma: 1 h: 86.06%, 24 h: 96.19%, 168 h: 93.15%). As limma allows for the generation of models that can incorporate more than one predictor variable, and for the application of empirical Bayes variance shrinkage that produces more robust findings, we report enriched proteins identified by limma within the main results only. To test if the proportion of significantly differentially abundant proteins was more than expected by chance (i.e., > 50%), we performed a one‐sided binomial exact test using base functions in R for each time point. To examine whether significantly differentially abundant proteins affected by both envenomation and time showed correlated abundance changes, we performed Pearson product–moment correlations using base functions in R between log_2_ fold change values for the envenomation effect and the time effect at 24 and 168 h. For binomial exact tests and correlation‐based tests, *p*‐values less than 0.05 were considered as statistically significant. In addition, we examined overlaps of shared proteins with significantly elevated and reduced abundance in response to envenomation across time points using the R package eulerr (v. 7.0.2; (Larsson 2024)). For additional data visualisation, we used the R packages ggplot2 (v. 3.5.1; (Wickham 2016)) and ggpubr (v. 0.6.0; (Kassambara 2023)).

### 2.6 Gene Ontology Term Enrichment Analysis

To determine biological processes influenced by the act of envenomation, we performed Gene Ontology (GO) term enrichment analyses using Fisher's exact tests (Benjamini‐Hochberg adjusted *p*‐values < 0.05) implemented in R using the package topGO (v. 2.54.0; (Alexa and Rahnenfuhrer 2023)) with the “weight01” algorithm. Due to the depauperate nature of the annotations assigned to non‐model organisms, such as bumblebees, we downloaded GO terms assigned to genes in the fruit fly *Drosophila melanogaster* and then transferred them to their homologues in the *B. terrestris* genome Bter_1.0 (Sadd et al. 2015). Genes with no annotated GO term or genes coding for proteins that lack homology with proteins of *D. melanogaster* were excluded. We then used this generated database to test for enrichment of GO terms for genes of interest. In total, we performed GO term enrichment analyses for: (1) all genes coding for identified protein groups found in the venom sac (protein group list of the MaxQuant output) to determine biological processes associated with proteins found in the venom sac; (2) and proteins that had elevated or reduced abundance post‐envenomation across each time point, respectively, using scripts from (Colgan et al. 2019). All GO enrichment analyses were conducted using a node‐size of 50.

## 3 Results

As an initial exploratory approach, we performed a principal component analysis to determine whether samples clustered based on treatment or time point. Using only proteins that were detected across all samples (i.e., no missing or imputed values), we found no strong separation of samples based on either treatment or time point at a global level (Supporting Information, Figure S2). To next understand how the venom sac proteome changes at specific time points (1, 24, and 168 h post‐envenomation), we performed pairwise comparisons of the envenomation treatment and control bumblebees at each time point using limma. Using proteins with imputed values, we found that envenomation was collectively associated with moderate changes in the venom sac proteome with between ~9% and 14% of the total identified proteins changing in abundance across the three time points examined. More specifically, we identified significantly differentially abundant proteins (SDAPs) (Moderated *t*‐test: BH‐adjusted *p*‐value < 0.05) at each time point (1 h: 190 SDAPs; 24 h: 292 SDAPs; 168 h: 236 SDAPs; Figure 2) with a significantly higher number of SDAPs (~65%–78% of all SDAPs) having reduced abundance in the venom sac post‐envenomation at all time points (Binomial exact tests: *p*‐value < 0.05; 1 h: 138 SDAPs, 24 h: 230 SDAPs, 168 h: 155 SDAPs) suggestive of depletion of specific proteins or reduced translational activity. Of this number, we found that certain proteins were also significantly changed in expression between time points (24 h = 28; 168 h = 70) but for these shared proteins, expression was negatively correlated (Pearson's product–moment correlation: *R* ≤ −0.68, *p*‐value < 1e−05; Supporting Information, Figure S3) indicating that changes in expression related to treatment were not fully explained by time.

**FIGURE 2:**
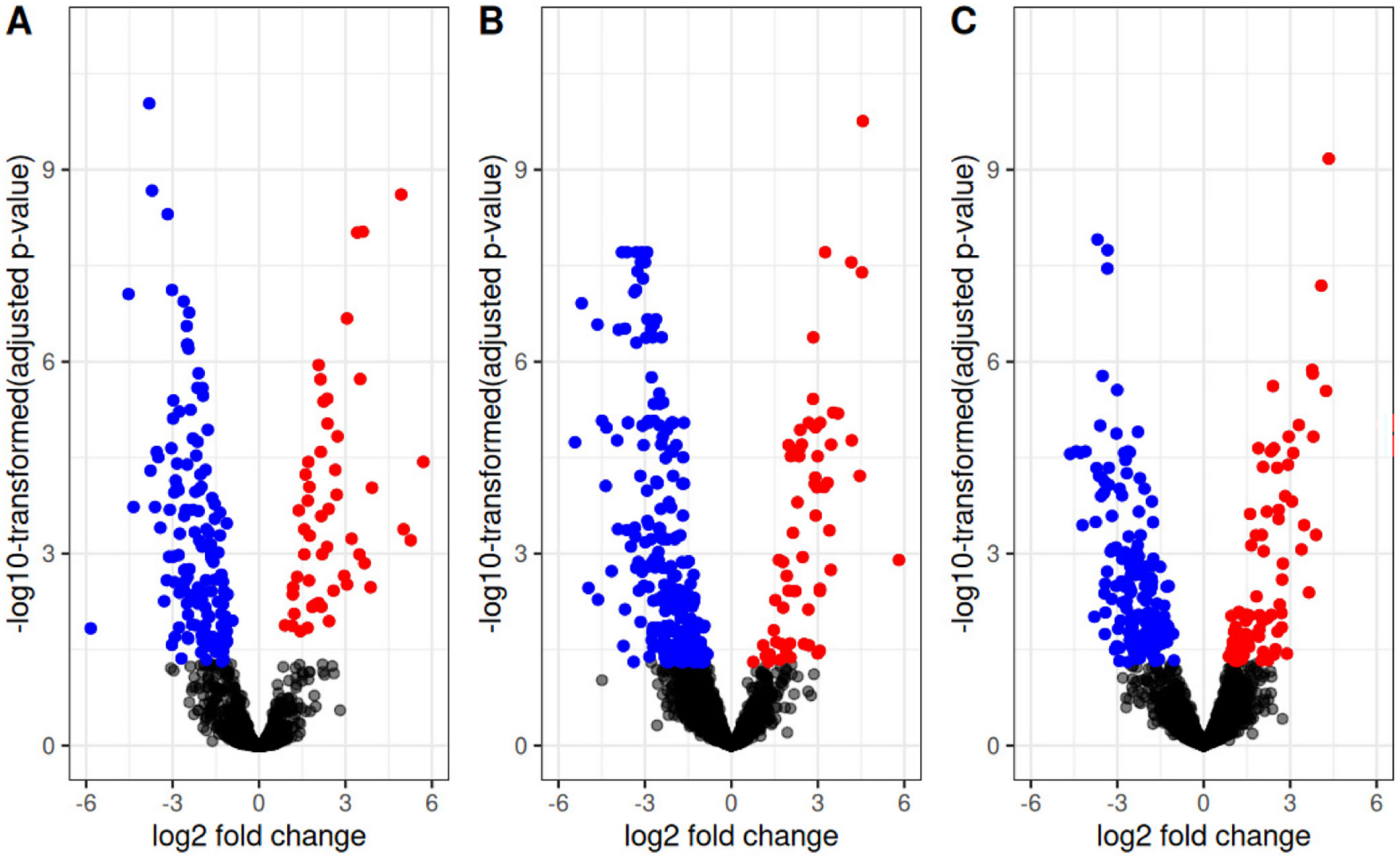
Envenomation leads to changes in protein abundance in the venom sac of *Bombus terrestris* workers at three different time points. Volcano plots showing the protein abundance differences (log_2_ fold change) between workers post‐envenomation relative to control workers at: (A) 1 h; (B) 24 h; and (C) 168 h post‐envenomation. Proteins with significantly (Moderated *t*‐test, Benjamini‐Hochberg‐adjusted *p*‐value < 0.05) reduced or elevated abundance post‐envenomation are individually coloured (blue = reduced abundance; red = elevated abundance; grey = unchanged).

To understand the biological processes that such proteins are involved in, we performed GO term enrichment analyses finding that genes coding for proteins reduced after all three time points were significantly enriched for “cytoplasmic translation” (GO:0002181), as well as for “protein‐RNA complex assembly” (GO:0022618) after 24 h (Fisher's exact test: BH‐adjusted *p*‐value < 0.05; Supporting Information, Table S2). Despite general patterns of reduced individual protein abundance in the venom sac post‐envenomation, we also found proteins that increased in abundance after venom usage (1 h: 52 SDAPs, 24 h: 62 SDAPs, 168 h: 81 SDAPs). There was no significant enrichment of biological process‐related GO terms for elevated proteins at any of the three time points (Fisher's exact test, BH‐adjusted *p*‐value > 0.05; Supporting Information, Table S2). Most reduced SDAPs were unique to individual time points with 352 SDAPs uniquely identified at one time point, 75 proteins shared between two time points and 7 proteins shared at all three time points. For elevated proteins post‐envenomation, there were 157 unique proteins at one time point and 19 proteins which were elevated at two time points (Supporting Information, Figure S4).

As our analyses revealed general reductions in protein abundance post‐envenomation, which may be expected due to venom use and depletion, we examined abundance changes of previously described venom proteins. We identified reduced expression (Moderated *t*‐test, BH‐adjusted < 0.05; Supporting Information, Table S3) within the envenomation group of prominent components of bumblebee venom, such as phospholipase A2‐like (XP_012170988.1) at 1 h and bombolitin/melittin (XP_020718386.1), phospholipase A2 (XP_012170987.1) and a hyaluronidase‐like protein (XP_012171522.1) at 24 h post‐envenomation, respectively. In contrast, no differences in expression levels for these proteins were observed at 168 h post‐envenomation between control and treatment bees (Moderated *t*‐test, BH‐adjusted *p*‐value > 0.05; Supporting Information, Table S3). While the majority of putative venom proteins had reduced expression, we identified one venom protein, a trehalase‐like protein (XP_003400853.1), with increased abundance at 24 h post‐envenomation.

## 4 Discussion

Many animals across different lineages use venom, mainly for predation and defence, highlighting the ecological and evolutionary importance of this biological innovation. Nevertheless, synthesis of venom and maintaining a venom system is energetically costly (Morgenstern and King 2013; Wigger et al. 2002; Evans et al. 2019). Given this energetic requirement (Morgenstern and King 2013; Wigger et al. 2002; Evans et al. 2019), the aim of this study was to examine the potential consequences at the molecular level associated with venom usage (envenomation) in *B. terrestris* workers, where venom is mainly used for individual and colony defence. Therefore, we performed a proteomic‐based analysis to understand how individual protein abundances within the venom sac, the primary reservoir of venom proteins, change for *B. terrestris* workers after envenomation, but also to determine whether envenomation leads to short‐term changes that may affect reuse. We found that envenomation results in changes in the venom sac proteome with relatively larger reductions in protein abundances, including prominent components of bumblebee venom, suggestive of potential costs at least at the molecular level. Our analysis also highlights a period required for venom protein abundances to recover compared to non‐envenomation controls, as evident by reduced abundances after 24 h post‐envenomation, which are recovered at least 1 week later, which may have consequences for short‐term potency and reuse.

During venom delivery, venomous organisms contract muscles of the venom reservoir to allow for the regulated release of venom to structures that facilitate delivery (Schendel et al. 2019). In our analysis of the proteins that changed after envenomation, the majority were reduced, including notable reductions of previously described venom components, which is in line with the possible depletion of stored venom proteins due to venom usage. Among the venom proteins reduced in the venom sac within 24 h, we identified phospholipase A2 and bombolitin, which are among the most abundant proteins in bumblebee venom (Choo et al. 2010). Phospholipase A2 induces inflammatory responses (Six and Dennis 2000) with bumblebee phospholipase A2 exhibiting apoptotic activity in mammals (Xin et al. 2009). Melittin, the honeybee's homologue of bombolitin, is the most abundant peptide in honeybee venom and is known to induce strong and painful reactions in target organisms (Six and Dennis 2000), which, given the close relationship, could also be the case for bombolitin. In addition, bombolitin enhances phospholipase A2 activity (Argiolas and Pisano 1985) suggesting an important role of both components in venom potency.

The observed short‐term reduction of venom proteins after envenomation may result in less potent venom which may not induce as much pain and be less toxic in their targets. For example, snakes may use dry bites to conserve venom usage (Pucca et al. 2020) or deliver lower dosages during venom regeneration (McCue 2006). The availability of less venom could lead to behavioural changes of the individual. Although not examined in the present study, there is evidence that some venomous animals are aware of their venom status resulting in distinct behaviours if less venom is available, such as in some snakes, which hunt smaller prey or use constriction after they used venom or when venom is limited (Schendel et al. 2019). In bumblebees, it could be that individuals are less likely or unable to appropriately defend the nest directly post‐envenomation. However, this individual cost in repeated defence may be offset or compensated for by nestmates, which can act cooperatively to deter colony‐level attacks. Further research is required in this aspect to understand cooperative group work in nest defences with respect to how it influences venom usage and regeneration.

Venom usage is often associated with regeneration time after use. Our analysis found reductions at two points within 24 h post‐envenomation, though venom proteins were observed to have similar abundance levels with control individuals after 7 days post‐envenomation. While our study design did capture three time points across a period of up to 168 h post‐envenomation, the recovery of venom reserves could occur at some point between 24 h and 168 h. Thus, future profiling of shorter‐term time points may be able to capture more immediate changes post‐envenomation. Our results nonetheless indicate that recovery of key venom components occurs within a week of envenomation. As it takes time to replenish the venom protein abundance, there are periods when venom cannot be used or when less venom may be available. Timing of venom regeneration varies across species with regeneration potentially taking up to several weeks (Boevé et al. 1995; Morgenstern and King 2013). Speed of regeneration is also influenced by metabolic rate. For example, the metabolic rate of the scorpion *Parabuthus transvaalicus* increases by 21%–40% in the first days of venom regeneration (Nisani et al. 2007, 2012), highlighting metabolic costs to replenish venom. While we did not directly measure metabolic activity, proteins involved in translation were differentially abundant immediately after envenomation, which may reflect investment of energetic resources to replenish used venom. However, as bumblebees are social insects, the recovery of venom proteins may not only be affected by metabolic costs but also by their social environment. For example, in the social acorn ant *Temnothorax longispinosus*, venom production was associated with social cues. Venom investment by an individual was linked to social parasite pressure whereby colonies that experienced a social parasite raid had more estimated venom per individual. In addition, venom investment after a raid was associated with the task that a single individual performs with foragers having more venom than nurses (Koenig and Moreau 2024). Thus, potential threats and social factors, such as a low number of individuals, may influence venom production and venom recovery of an individual to ensure the colony's viability. Bumblebees typically use venom to defend their nests, and the colony usually consists of numerous individuals that are able to contribute to the collective nest defence which could enable the investment in venom, including the time required for replenishment, to be distributed among nestmates. In our study, workers were kept in small social groups which may lead to a faster venom recovery to be capable of defending in the event of an attack. Additionally, as the number of individuals slightly decreased within each box due to sampling over the course of the experiment, the lower number of individuals over time could also contribute to expression changes in the venom sac.

During venom regeneration, energetic costs could be compensated by increased food consumption. At 24 h post‐envenomation, the venom associated protein trehalase was elevated in the venom sac. Trehalase is an enzyme also found in the haemolymph of insects which hydrolyses trehalose, the primary storage sugar in insects found in the haemolymph and in venom glands (Kote et al. 2019), into glucose and is associated with the hunger state and appetite regulation in honeybees (Blatt and Roces 2001, 2002; Akülkü et al. 2021). Low trehalose levels correspond with an increase in octopamine levels in the honeybee brain leading to elevated appetite levels (Blatt and Roces 2001, 2002; Akülkü et al. 2021). Thus, elevated trehalase levels in response to envenomation could lead to a higher availability of glucose, which is the main energy resource in insects, and simultaneously to lower trehalose levels resulting in an enhanced appetite to compensate for energetic costs for venom regeneration. As bumblebees use venom for defence and do not offset energetic costs by nutritional gain from successful prey capture and consumption, this could be a mechanism to ensure offsetting energetic costs. Higher food consumption can offset the negative energetic effects of immune stimulation in *B. terrestris (*Moret and Schmid‐Hempel 2000; Tyler et al. 2006) and, therefore, may potentially represent a mechanism to compensate for the induction of metabolically costly activities. In our experiments, small micro‐colonies with low worker density were used that were provided with ample food, which could expedite recovery in at least 1 week, whereby in the natural environment, under restricted resources, recovery time may take longer. Future studies would benefit from examining how venom regeneration is affected by resource limitation.

## 5 Conclusions

Venom plays an essential role in the defence of social insects, such as bumblebees, yet usage can result in consequences observable at different biological levels. Using proteomic‐based profiling of an organ fundamental in the storage and usage of venom, we show that envenomation and subsequent regeneration are associated with changes in protein abundance, with the majority being reduced, including known venom proteins. Our findings also indicate a time period greater than 1 day required for venom recovery, which may have implications for repeated use as well as the potency of bumblebee venom during regeneration. Future work will benefit from increased sample sizes, allowing for higher‐resolution capture of both colony‐level and individual variation, as well as the inclusion of measurements of the venom sac, venom gland and body size, which may be correlated to protein abundance. Additionally, direct examination of venom products, including bioassays, may provide insights into shifts in potency after envenomation. Finally, investigating how venom usage may affect other life history traits, such as longevity or reproduction, is of particular interest given venom can fulfil a diverse range of functions from hygiene and social immunity to individual and colony defence. Collectively, our findings demonstrated molecular consequences of venom use on the whole venom sac, an organ essential for venom delivery, as well as the period required for venom recovery which represents a period of potentially increased vulnerability.

## Author Contributions

**Safira Moog:** conceptualization (equal), data curation (lead), formal analysis (lead), investigation (lead), methodology (lead), project administration (equal), visualization (lead), writing – original draft (lead), writing – review and editing (lead). **Mario Dejung:** data curation (supporting), formal analysis (supporting), methodology (supporting). **Amitkumar Fulzele:** data curation (supporting), methodology (supporting). **James C. Carolan:** formal analysis (supporting), methodology (supporting). **Thomas J. Colgan:** conceptualization (equal), formal analysis (supporting), funding acquisition (equal), investigation (supporting), methodology (supporting), project administration (equal), resources (lead), supervision (lead), writing – original draft (supporting), writing – review and editing (equal).

## Funding

This study was funded in part by Stufe‐I inneruniversity funds awarded to T.J.C. while the project used a Q Exactive Plus System funded by the German Research Foundation (DFG Project #240874965). We would like to thank the German Federal Environmental Foundation (Deutsche Bundesstiftung Umwelt, DBU) for supporting S.M. with a doctoral scholarship (20023/011).

## Ethics Statement

All animal procedures were conducted according to the principles of the Directive 2010/63/EU on the protection of animals used for scientific purposes which declares that no formal authorisation for this experiment using bees was required. However, the experimental design and conduction of this study were developed to minimise stress and to use the minimum viable number of animals to allow us to reach our research objectives, as well as to ensure that handling and maintenance of the bees were appropriately performed throughout the experiment.

## Conflicts of Interest

The authors declare no conflicts of interest.

## Supporting information


**Figure S1:** Temporal changes in protein abundance in the venom sac of *Bombus terrestris* workers. Volcano plots showing the protein abundance differences (log_2_ fold change) between workers in response to time at: (A) 24 h in comparison to 1 h; and (B) at 168 h in comparison to 1 h. Proteins with significantly (Moderated *t*‐test, Benjamini‐Hochberg‐adjusted *p*‐value < 0.05) reduced or elevated abundance post‐treatment are individually coloured (blue = reduced abundance; red = elevated abundance; grey = unchanged).
**Figure S2:** Principal component analysis of the venom sac proteome of bumblebee workers. A scatterplot displaying the first two principal components calculated from a principal component analysis (PCA) based on label‐free quantification (LFQ) intensity values for identified venom sac proteins, which were detected across all samples before imputation (*n* = 682 proteins). The first two components (Principal component 1 (PC1), and principal component 2 (PC2)) are provided on the *x*‐ and *y*‐axis, respectively, which collectively, explain approximately 50% of the variance. PC2 slightly separate certain individuals from the later envenomation treatments (24 and 168 h post‐treatment). Each point represents an individual sample and each treatment per time point is indicated by an individual colour.
**Figure S3:** Proteins that change in the venom sac of *Bombus terrestris* workers in response to envenomation and time show a negative correlation. Scatterplots showing the protein abundance differences (log_2_ fold change) for significantly abundant proteins for: (A) at 24 h post‐envenomation in comparison to 1 h; and (B) 168 h post‐envenomation in comparison to 1 h post‐envenomation. Proteins with significant abundance changes (Moderated *t*‐test, Benjamini‐Hochberg‐adjusted *p*‐value < 0.05) in response to both, envenomation and time, are coloured in orange. For both time points, Pearson's product–moment‐based correlations indicate negative correlations in terms of the direction of change for proteins affected by both envenomation and time.
**Figure S4:** Overlap of significantly reduced or elevated venom sac proteins of *Bombus terrestris* workers in response to envenomation across all time‐points. Euler plot displaying the number of: (A) reduced differentially abundant venom sac proteins at all time‐points; and (B) elevated differentially abundant venom sac proteins at all time‐points. Each time point is represented by an individual colour (1 h = white; 24 h = grey; 168 h = blue).

**Table S1a:** Proteins identified from the venom sac of *Bombus terrestris* workers 1, 24 and 168 h post‐envenomation and of workers 1, 24 and 168 h post‐control treatment (no envenomation). Protein IDs, genomic loci, mass spectrometry characteristics and log_2_ transformed LFQ intensity values are provided.
**Table S1b:** Proteins identified from the venom sac of *Bombus terrestris* workers 1 h post‐envenomation and of workers 1 h post‐control treatment (no envenomation). Protein IDs, genomic loci, mass spectrometry characteristics and log_2_ transformed LFQ intensity values are provided.
**Table S1c:** Proteins identified from the venom sac of *Bombus terrestris* workers 24 h post‐envenomation and of workers 24 h post‐control treatment (no envenomation). Protein IDs, genomic loci, mass spectrometry characteristics and log_2_ transformed LFQ intensity values are provided.
**Table S1d:** Proteins identified from the venom sac of *Bombus terrestris* workers 168 h post‐envenomation and of workers 168 h post‐control treatment (no envenomation). Protein IDs, genomic loci, mass spectrometry characteristics and log_2_ transformed LFQ intensity values are provided.
**Table S1e:** Venom‐associated proteins with peptide‐spectrum‐matches value ≥ 20 from the previously identified *Bombus terrestris* venom proteome by Van Vaerenbergh et al. (2015) are provided (venom proteins); provided are the NCBI RefSeq protein accession IDs and the description of the protein. Proteins highlighted in yellow were identified in the venom sac of *B. terrestris* workers.

**Table S2a:** Gene Ontology term enrichment for elevated venom sac proteins 1 h post‐envenomation of *Bombus terrestris* workers using a Fisher's exact test (Benjamini Hochberg *p* < 0.05) with the ‘weight01’ algorithm (node size = 50) using the R package topGO. Here, GO Ids and terms assigned to “biological processes”, and the statistical output of the Fisher's exact test are provided.
**Table S2b:** Gene Ontology term enrichment for reduced venom sac proteins 1 h post‐envenomation of *Bombus terrestris* workers using a Fisher's exact test (Benjamini Hochberg *p* < 0.05) with the ‘weight01’ algorithm (node size = 50) using the R package topGO. Here, GO Ids and terms assigned to “biological processes”, and the statistical output of the Fisher's exact test are provided.
**Table S2c:** Gene Ontology term enrichment for elevated venom sac proteins 24 h post‐envenomation of *Bombus terrestris* workers using a Fisher's exact test (Benjamini Hochberg *p* < 0.05) with the ‘weight01’ algorithm (node size = 50) using the R package topGO. Here, GO Ids and terms assigned to “biological processes”, and the statistical output of the Fisher's exact test are provided.
**Table S2d:** Gene Ontology term enrichment for reduced venom sac proteins 24 h post‐envenomation of *Bombus terrestris* workers using a Fisher's exact test (Benjamini Hochberg *p* < 0.05) with the ‘weight01’ algorithm (node size = 50) using the R package topGO. Here, GO Ids and terms assigned to “biological processes”, and the statistical output of the Fisher's exact test are provided.
**Table S2e:** Gene Ontology term enrichment for elevated venom sac proteins 168 h post‐envenomation of *Bombus terrestris* workers using a Fisher's exact test (Benjamini Hochberg *p* < 0.05) with the ‘weight01’ algorithm (node size = 50) using the R package topGO. Here, GO Ids and terms assigned to “biological processes”, and the statistical output of the Fisher's exact test are provided.
**Table S2f:** Gene Ontology term enrichment for reduced venom sac proteins 168 h post‐envenomation of *Bombus terrestris* workers using a Fisher's exact test (Benjamini Hochberg *p* < 0.05) with the ‘weight01’ algorithm (node size = 50) using the R package topGO. Here, GO Ids and terms assigned to “biological processes”, and the statistical output of the Fisher's exact test are provided.
**Table S2g:** Gene Ontology term enrichment for venom sac proteins post‐envenomation of *Bombus terrestris* workers using a Fisher's exact test (Benjamini Hochberg *p* < 0.05) with the ‘weight01’ algorithm (node size = 50) using the R package topGO. Here, GO Ids and terms assigned to “biological processes”, and the statistical output of the Fisher's exact test are provided.

**Table S3a:** LogFC values calculated using the R package limma for proteins identified from the venom sac of *Bombus terrestris* workers 1 h post‐envenomation and of workers 1 h post‐control treatment (no envenomation). LogFC values, statistical output of moderated *t*‐tests, the adjusted *p*‐value (Benjamini‐Hochberg), and the Protein Ids are provided.
**Table S3b:** LogFC values calculated using the R package limma for proteins identified from the venom sac of *Bombus terrestris* workers 24 h post‐envenomation and of workers 24 h post‐control treatment (no envenomation). LogFC values, statistical output of moderated *t*‐tests, the adjusted *p*‐value (Benjamini‐Hochberg), and the Protein Ids are provided.
**Table S3c:** LogFC values calculated using the R package limma for proteins identified from the venom sac of *Bombus terrestris* workers 168 h post‐envenomation and of workers 168 h post‐control treatment (no envenomation). LogFC values, statistical output of moderated *t*‐tests, the adjusted *p*‐value (Benjamini‐Hochberg), and the Protein Ids are provided.
**Table S3d:** LogFC values calculated using the R package limma for proteins identified from the venom sac of *Bombus terrestris* workers post‐envenomation and of workers post‐control treatment (no envenomation). Here, differences were calculated for the time point at 24 h after envenomation in comparison to the control at 1 h. LogFC values, statistical output of moderated *t*‐tests, the adjusted *p*‐value (Benjamini‐Hochberg), and the Protein Ids are provided.
**Table S3e:** LogFC values calculated using the R package limma for proteins identified from the venom sac of *Bombus terrestris* workers post‐envenomation and of workers post‐control treatment (no envenomation). Here, differences were calculated for the time point at 168 h after envenomation in comparison to the control at 1 h. LogFC values, statistical output of moderated *t*‐tests, the adjusted *p*‐value (Benjamini‐Hochberg), and the Protein Ids are provided.

## Data Availability

Raw mass spectrometry data are publicly available via the PRIDE database (PXD066016; https://www.ebi.ac.uk/pride/archive?keyword=%28PXD066016%29). Additional methods, results, data, and analysis code are available in the electronic Supporting Information.
