# Supplementary material for: Envenomation Leads to Venom Protein Reduction and a Recovery Period in Bumblebee Workers
Source: Ecol Evol. 2026 Nov 5;16(11):e74629. doi: 10.1002/ece3.74629 (PMC13740374; doi:10.1002/ece3.74629)
Supplement: Supplementary file 1 [file ECE3-16-e74629-s004.docx]

**Supplementary Information**

**Proteomic changes in the venom sac that occur with time**

As proteins associated with venom usage may also be affected by natural changes due to time, we assessed proteins that changed in response to time using a linear model fit with limma. After 24h, 104 proteins had changed in abundance while at 168h, we found 441 SDAPs that had changed (Moderated t-test: BH-adjusted p-value < 0.05). In comparison to 1h post venom usage, most proteins were reduced at 24h (74 reduced, 20 elevated) and 168h (366 reduced, 75 elevated), respectively (Figure S1). Similarly, in comparison to the 1h control group, there was no strong overlap between the effect of treatment and time, meaning most proteins changing by time were independent of treatment, i.e., venom usage (24h: 340 proteins changed by either treatment or time, 28 proteins changed by both treatment and time; 168h: 537 changed by either treatment or time, 70 proteins changed by both treatment and time). Proteins that were significantly differentially abundant by both time and envenomation showed a weak negative correlation (24h: *R* = -0.78, p-value = 1.1e-06; 168h: *R* = -0.68, p-value = 7.47e-11; Figure S3), with proteins that increase in abundance in response to treatment tending to decrease over time. Lastly, we found two venom-associated proteins which showed reduced abundance over time at 168h: a probable chitinase 10 isoform X1 protein (XP_020721052.1) and a beta-hexosaminidase subunit beta-like protein (XP_003397815.1).

**Figure S1. Temporal changes in protein abundance in the venom sac of *Bombus terrestris* workers.** Volcano plots showing the protein abundance differences (log_2_ fold change) between workers in response to time at: **(A)** 24h in comparison to 1h; and **(B)** at 168h in comparison to 1h. Proteins with significantly (Moderated t-test, Benjamini-Hochberg-adjusted p-value < 0.05) reduced or elevated abundance post-treatment are individually coloured (blue = reduced abundance; red = elevated abundance; grey = unchanged).

**Proteomic changes in the bumblebee venom sac as determined by Perseus**

As a complementary approach to determine differentially abundant proteins through the R package limma, we also used Perseus to examine abundance changes in the venom sac proteome post-envenomation. Across all three time points, 5-8% of the total identified proteins were identified as significantly differentially abundant proteins (SDAPs) (1h: 165 SDAPs; 24h: 105 SDAPs; 168h: 73 SDAPs; Welch’s t-test: Benjamini Hochberg-adjusted p-value < 0.05). At all time points post-envenomation, envenomation was associated with a significantly higher number of SDAPs (76-79% of all SDAPs) having reduced abundance in the venom sac, which was a significant trend (Binomial exact tests: p-value < 0.05; 1h: 116 SDAPs, 24h: 81 SDAPs, 168h: 56 SDAPs).­

To understand functional enrichment of Perseus-identified SDAPs, we performed a Gene Ontology (GO) term enrichment analyses revealing that genes coding for proteins which were reduced after 1h and 24h, respectively, were significantly enriched for “cytoplasmic translation” (GO:0002181) (Fisher’s exact test: BH-adjusted p-value < 0.05). After 168h post-envenomation, there was no significant enrichment of GO terms for reduced SDAPs (Fisher’s exact test, BH-adjusted p-value > 0.05). We found fewer proteins that increased in abundance after venom usage (1h: 37 SDAPs, 24h: 22 SDAPs, 168h: 15 SDAPs). For proteins with elevated abundance, there was no significant enrichment of GO terms assigned to biological processes at any of the three time points (Fisher’s exact test, BH-adjusted p-value> 0.05).

**Principal component analysis**

To first understand how the venom sac proteome changes with envenomation status and time, we performed a principal component analysis (PCA) using only proteins detected based on non-imputed values across all treatments (control and envenomation) and time points (1h, 24h and 168h post-treatment). Our exploratory approach did not reveal strong proteome-wide abundance changes with samples not generally clustering based on time point or treatment. The first principal component (PC1) explained 31.93% of the variance, separating a single sample from the others. In comparison, the second principal component (PC2), explaining 17.95% of the variance, showed weak separation of certain individuals from the later envenomation treatments (24h and 168h post-treatment; Figure S2).

**Figure S2. Principal component analysis of the venom sac proteome of bumblebee workers**. A scatterplot displaying the first two principal components calculated from a principal component analysis (PCA) based on label-free quantification (LFQ) intensity values for identified venom sac proteins, which were detected across all samples before imputation (n= 682 proteins). The first two components (Principal component 1 (PC1), and principal component 2 (PC2)) are provided on the x- and y-axis, respectively, which collectively, explain approximately 50% of the variance. PC2 slightly separate certain individuals from the later envenomation treatments (24h and 168h post-treatment). Each point represents an individual sample and each treatment per time point is indicated by an individual colour.

**Figure S3. Proteins that change in the venom sac of *Bombus terrestris* workers in response to envenomation and time show a negative correlation.** Scatterplots showing the protein abundance differences (log_2_ fold change) for significantly abundant proteins for: **(A)** at 24h post-envenomation in comparison to 1h; and **(B)** 168h post-envenomation in comparison to 1h post-envenomation. Proteins with significant abundance changes (Moderated t-test, Benjamini-Hochberg-adjusted p-value < 0.05) in response to both, envenomation and time, are coloured in orange. For both time points, Pearson's product-moment-based correlations indicate negative correlations in terms of the direction of change for for proteins affected by both envenomation and time.

**Figure S4. Overlap of significantly reduced or elevated venom sac proteins of *Bombus terrestris* workers in response to envenomation across all time-points.** Euler plot displaying the number of**: (A)** reduced differentially abundant venom sac proteins at all time-points; and **(B)** elevated differentially abundant venom sac proteins at all time-points. Each time point is represented by an individual colour (1h = white; 24h = grey; 168h = blue).
